# Re-evaluation of *Zospeumschaufussi* von Frauenfeld, 1862 and *Z.suarezi* Gittenberger, 1980, including the description of two new Iberian species using Computer Tomography (CT) (Eupulmonata, Ellobioidea, Carychiidae)

**DOI:** 10.3897/zookeys.835.33231

**Published:** 2019-04-04

**Authors:** Adrienne Jochum, Carlos E. Prieto, Marian Kampschulte, Gunhild Martels, Bernhard Ruthensteiner, Marko Vrabec, Dorian D. Dörge, Anton J. de Winter

**Affiliations:** 1 Naturhistorisches Museum der Burgergemeinde Bern (NMBE), CH-3005 Bern, Switzerland Naturhistorisches Museum der Burgergemeinde Bern Bern Switzerland; 2 Departamento de Zoología y Biología Cellular Animal, Universidad del País Vasco (UPV-EHU), 48080 Bilbao, Spain Universidad del País Vasco Bilbao Spain; 3 Universitätsklinikum Giessen und Marburg GmbH-Standort Giessen, Center for Radiology, Dept. of Radiology, 35385 Giessen, Germany Universitätsklinikum Gießen und Marburg Gießen Germany; 4 Department of Experimental Radiology, Justus-Liebig University Giessen, Biomedical Research Center Seltersberg (BFS), 35392 Giessen, Germany Justus-Liebig University Gießen Gießen Germany; 5 Section Evertebrata varia, Zoologische Staatssammlung München (ZSM), 81247 München, Germany Zoologische Staatssammlung München München Germany; 6 Department of Geology, Faculty of Natural Sciences and Engineering, University of Ljubljana,1000 Ljubljana, Slovenia University of Ljubljana Ljubljana Slovenia; 7 Institute for Ecology, Evolution and Diversity; Senckenberg Biodiversity and Climate Research Center (BiK-F); Senckenberg Gesellschaft für Naturforschung (SGN), Goethe-University (GU), 60438 Frankfurt/M., Germany Goethe-University Frankfurt am Main Germany; 8 Naturalis Biodiversity Center, P.O. Box 9517, NL 2300 RA Leiden, The Netherlands Naturalis Biodiversity Center Leiden Netherlands

**Keywords:** Cave-dwelling species, microgastropods, shell variability, subterranean land snail

## Abstract

The present study aims to clarify the confused taxonomy of *Z.schaufussi* von Frauenfeld, 1862 and *Zospeumsuarezi* Gittenberger, 1980. Revision of Iberian *Zospeum* micro snails is severely hindered by uncertainties regarding the identity of the oldest Iberian *Zospeum* species, *Z.schaufussi* von Frauenfeld, 1862. In this paper, we clarify its taxonomic status by designating a lectotype from the original syntype series and by describing its internal and external shell morphology. Using SEM-EDX, we attempt to identify the area of the type locality cave more precisely than “a cave in Spain”. The shell described and illustrated by Gittenberger (1980) as *Z.schaufussi* appears not to be conspecific with the lectotype shell, and is considered a separate species, *Z.gittenbergeri* Jochum, Prieto & De Winter, **sp. n.**

*Zospeumsuarezi* was described from various caves in NW Spain. Study of the type material reveals that these shells are not homogenous in shell morphology. The holotype shell of *Z.suarezi* is imaged here for the first time. The paratype shell, illustrated by Gittenberger (1980) from a distant, second cave, is described as *Zospeumpraetermissum* Jochum, Prieto & De Winter, **sp. n.** The shell selected here as lectotype of *Z.schaufussi*, was also considered a paratype of *Z.suarezi* by Gittenberger (1980). Since this specimen is morphologically very similar to topotypic shells of *Z.suarezi*, the latter species is considered a junior synonym of *Z.schaufussi* (**syn. n.**). The internal shell morphology of all these taxa is described and illustrated using X-ray Micro Computer Tomography (Micro-CT).

## Introduction

**Figure 1.**
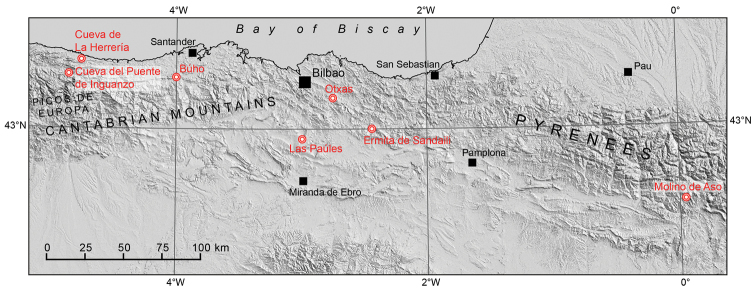
Map indicating geographic position of type locality caves of described species of *Zospeum* in northern Spain. From left to right: Cueva del Puente de Inguanzo (*Z.gittenbergeri* sp. n., *Z.praetermissum* sp. n.), Cueva de La Herrería (*Z.percostulatum*), Cueva de Búho (*Z.suarezi*, syn. n. of *Z.schaufussi*), Cueva de Las Paúles (*Z.zaldivarae*), Cueva de Otxas (*Z.biscaiense*), Cueva de la Ermita de Sandaili (*Z.vasconicum*) and Cueva Molino de Aso (*Z.bellesi*). Source of DEM data: ALOS Global Digital Surface Model (AW3D30), JAXA.

The Cantabrian-Pyrenean Region, encompassing 500 × 50 km of the northwestern part of the Iberian Peninsula, harbours a remarkable diversity of the cave-dwelling, land snail genus *Zospeum*. A number of species have been formally described. The oldest available name for a Spanish *Zospeum* species is *Z.schaufussi* von Frauenfeld, 1862. Since then, six more Spanish species have been described, viz., *Zospeumbellesi* Gittenberger, 1973; *Z.suarezi* Gittenberger, 1980; *Z.biscaiense* Gómez & Prieto, 1983; *Z.vasconicum* Prieto, De Winter, Weigand, Gómez & Jochum, 2015; *Z.zaldivarae* Prieto, De Winter, Weigand, Gómez & Jochum, 2015, and recently *Z.percostulatum* Alonso, Prieto, Quiñonero-Salgado & Rolán, 2018 (Fig. 1).

Insufficient knowledge, causing doubts about the identity of two of these species, *Z.schaufussi* and *Z.suarezi*, has blocked further descriptions of this potentially very speciose radiation in a region where many caves are inhabited by two or three different morphotypes (Alonso et al. 2018).

*Zospeumschaufussi* was the first *Zospeum* species reported from Spain. Detailed information about its provenance is lacking while its description is insufficient according to today’s standards. When Gittenberger (1980) studied von Frauenfeld’s original material, he could not accept the available shells as syntypes of *Z.schaufussi*, because the damaged shells possess distinct barriers within the body whorl, the absence of which was mentioned as a specific character by von Frauenfeld (1862). Instead, he described and illustrated a shell from Cueva [del Puente] de Inguanzo near Inguanzo (Asturias) as *Z.schaufussi*. However, he did not formalize his view by selection of a neotype. In this paper, we select the single, undamaged original syntype shell as lectotype of *Z.schaufussi* and provide a re-description of this previously unclear taxon.

When Gittenberger (1980) received *Zospeum* material from various northern Spanish caves, only two other Iberian species, *Z.bellesi* Gittenberger, 1973 and *Z.schaufussi* were known. Since most of the shells appeared to be different from the latter two species, Gittenberger (1980) described this material as a new species, *Z.suarezi* Gittenberger, 1980, of which the holotype derived from the Cueva del Búho, Puente Viesgo (Cantabria). The new species, *Z.suarezi*, was illustrated by a drawing of a paratype shell from another cave, Cueva [del Puente] Inguanzo near Inguanzo (Asturias). These two caves are separated by a distance of 70 km. Illustrations of shells from the type cave have never been published.

Subsequent to its description, *Z.suarezi* was reported from Bizkaia, Burgos and Cantabria (e.g. Altonaga et al. 1994; Weigand et al. 2013). Weigand et al. (2013) attributed four populations (lineages Z13–Z16) to *Z.suarezi*. These populations showed significant variability in some genetic markers, but the provided images (BOLD database) of the now molecularly-processed voucher specimens, preclude study of their shell morphologies in sufficient detail. Later examination of additional material from these caves revealed ample variability in shell morphology, casting doubt as to the conspecificity of the populations used in this molecular study (unpublished results). Adding more confusion to the situation, a shell recently designated as *Z.suarezi* from Navarra, more than 200 km from the type cave, was illustrated in an authoritative guide to the land snails of the Iberian Peninsula (Cadevall and Orozco 2016). Whether these populations indeed belong to *Z.suarezi* is uncertain. Obviously, the identity of *Z.suarezi* needs clarifying.

Re-examination of the original material of *Z.suarezi* in RMNH Leiden, revealed that the holotype shell and the illustrated paratype shell exhibit subtle, but consistent differences in external morphology, which could be corroborated by study of the internal shell using x-ray Micro Computer Tomography (Micro-CT). In this paper, we re-describe and illustrate the shell morphology of *Z.suarezi* from the locus typicus. In addition, we describe the illustrated paratype shell as the holotype of a new species. Gittenberger (1980) also included the only undamaged syntype of *Z.schaufussi* from the von Frauenfeld collection in Vienna as paratype of *Z.suarezi*. This raises the question of their taxonomic relationship, which we address in this work.

## Materials and methods

Material studied is housed in the following collections:

**MHNG** Muséum d’Histoire Naturelle Genève, Geneva, Switzerland

**NHMW** Naturhistorisches Museum Wien, Vienna, Austria

**RMNH**Naturalis Biodiversity Center (formerly RijksMuseum van Natuurlijke Historie), Leiden, The Netherlands

Maps were produced with the freely available QGIS software (QGIS Development Team 2018). For shoreline vector data, we used the GSHHG database (Wessel and Smith 1996).

We emphasize that the RMNH catalog numbers, originally documented in Gittenberger (1980 fig. 2), partly differ from the ones nowadays employed in the RMNH collection. These were later changed because some numbers had been accidentally issued twice. For example, the *Z.suarezi* paratype lot, RMNH 55389, (Cueva del Búho, Puente Viesgo) was stated in Gittenberger (1980) as RMNH 55384 shells (see Table 1). We use the current catalog numbers here and list Gittenberger’s (1980) notation in square brackets.

According to Emmanuel Tardy, curator at the MHNG, who imaged Gittenberger’s (1980) designated paratype material before it got lost (in the mail) in January 2017, Gittenberger’s data (1980) corresponded to two lots: MNHG 96219 (ex. 978.363), which consisted of one vial containing two gelatin capsules with 4 individuals out of the 5 specimens Gittenberger (1980) assessed, separated into two different capsules (Fig. 5G–R). A second lot, MHNG 96220 (ex. 978.364), contained one shell from Cueva de Los Quesos (Fig. 5A–F).

Additionally, as paratypes, Gittenberger (1980) included shells from still other caves, such as the Cueva de Ernialde (= Hernialde) (NHMW MOL75000-E48.815) (Fig. 6).

**Table 1.** Shell measurements in mm (for methodology, see Jochum et al. 2015, fig. 1) of *Zospeumschaufussi*, *Z.praetermissum* and *Z.gittenbergeri*. Most shells of *Z.schaufussi* are type material of *Z.suarezi* Gittenberger, 1980, from the Cueva del Búho. Collection numbers are those presently used in RMNH, some differ from those used in Gittenberger (1980). Abbreviations: SH, shell height; SD, shell diameter; HLW, height of last whorl; PH, peristome height; PD, peristome diameter; W, number of whorls (counted as in Kerney and Cameron 1979); CT, coiling tightness W:*ln*SH (Emberton 2001).

	collection	SH	SD	HLW	PH	PD	W	SH/SD	HLW/SH	PH/SH	PH/PD	CT
*** Z. schaufussi ***												
**lectotype**	NHMW 71837	1.30					6.00					2.34
*** Z. suarezi ***												
**holotype**	RMNH.MOL.55383	1.21	0.78	0.62	0.42	0.42	6.00	1.55	0.51	0.35	1.00	2.41
**paratype**	RMNH.MOL.55384	1.07	0.71	0.56	0.40	0.40	5.55	1.51	0.52	0.37	1.00	2.34
**paratype**	RMNH.MOL.55384	1.20	0.73	0.66	0.43	0.41	6.00	1.64	0.55	0.36	1.05	2.41
**paratype**	RMNH.MOL.55384	1.00	0.64	0.56	0.37	0.35	5.50	1.56	0.56	0.37	1.06	2.39
**paratype**	RMNH.MOL.55384	0.99	0.67	0.56	0.40	0.39	5.15	1.47	0.57	0.40	1.03	2.25
**paratype**	RMNH.MOL.55384	1.04	0.70	0.59	0.36	0.40	5.55	1.48	0.56	0.34	0.89	2.37
**paratype**	RMNH.MOL.55384	1.20	0.70	0.62	0.33	0.39	6.20	1.72	0.51	0.27	0.83	2.49
**paratype**	RMNH.MOL.55384	1.11	0.74	0.59	0.42	0.41	5.90	1.50	0.53	0.37	1.00	2.45
**paratype**	RMNH.MOL. 55390	0.99	0.62	0.53	0.35	0.35	5.4	1.60	0.54	0.35	1.00	2.36
**paratype**	RMNH.MOL. 55390	1.04	0.70	0.59	0.36	0.4	5.5	1.49	0.57	0.35	0.90	2.35
	mean/median	1.08	0.70	0.59	0.38	0.39	5.68	1.53	0.54	0.36	1.00	2.37
	min	0.99	0.62	0.53	0.33	0.35	5.15	1.47	0.51	0.34	0.83	2.25
	max	1.30	0.78	0.66	0.43	0.42	6.2	1.72	0.57	0.40	1.06	2.49
***Z.praetermissum* sp. n.**												
**holotype**	RMNH.MOL.55391	1.08	0.75	0.67	0.41	0.42	4.60	1.44	0.62	0.38	0.99	1.93
**paratype**	RMNH.MOL.339954	1.07	0.71	0.64	0.39	0.41	4.85	1.52	0.59	0.37	0.97	2.04
**paratype**	RMNH.MOL.339954	1.21	0.76	0.71	0.40	0.45	5.20	1.58	0.58	0.33	0.90	2.09
	mean/median	1.12	0.74	0.67	0.40	0.42	4.88	1.52	0.59	0.37	0.97	2.04
***Z.gittenbergeri* sp. n.**												
**holotype**	RMNH.MOL.234166	1.49	0.92	0.89	0.53	0.58	5.50	1.62	0.59	0.35	0.90	2.03

### Image acquisition

**Digital Images and measurements.** Images were taken via a Leica DFC420 digital camera attached to a Leica M165C stereo microscope using Leica LAS V4.4 software.

Shell measurements were made on digital images as described in Jochum et al. (2015, fig. 1). The number of whorls of each shell was counted according to the method described in Kerney and Cameron (1979).

**Micro-CT.** Internal shell morphologies were accessed using different micro-CT systems. The lectotype shell of *Z.schaufussi* (NHMW 71837) was imaged and processed in animated video format at RJL Micro & Analytic GmbH, Karlsdorf-Neuthard, Germany using the system SkyScan 1172 (Bruker MikroCT, Kontich, Belgium). The scanner is equipped with a sealed micro focus X-ray source and an 11 Mpx CCD detector. The specimen was scanned with 4 µm pixel size in rotation steps of 0.6 ° at 59 kV tube voltage and 167 µA tube current during a 360 ° rotation. Reconstruction with cross sectional images was performed using a modified Feldkamp cone-beam reconstruction algorithm. The animated video was generated using a direct volume rendering method implemented in the software, CTvox.

Other *Zospeum* shells, except for the CT-imaged paratype of *Z.suarezi* (RMNH.MOL.55389 [55384] (Fig. 12) and the holotype of *Z.gittenbergeri* sp. n. (RMNH.MOL.234166) (Fig. 14), were imaged using a SkyScan 2011 (Bruker MicroCT, Kontich, Belgium) at the Department of Experimental Radiology, Justus-Liebig University Biomedical Research Center Seltersberg (BFS), Giessen, Germany. The shells were scanned 185 ° around their vertical axis in rotation steps of 0.23 ° at 80 kV tube voltage and 120 μA tube current. Reconstruction was performed using the Feldkamp cone beam reconstruction algorithm. Image resolution was 1.75 μm isotropic voxel side length with a grey scale resolution of 8 bit. Digital image post processing and visualization (maximum intensity projection – MIP, volume compositing and summed voxel projection) were displayed using the ANALYZE software package (ANALYZE 11.0, Mayo Clinic, Rochester, MN, USA).

*Zospeumgittenbergeri* sp. n. (RMNH.MOL.234166) was scanned at the Zoologische Staatssammlung München with a Phoenix Nanotom m (GE Measurement & Control, Wunstorf, Germany) cone beam CT scanner at a voltage of 80 kV and a current of 325 mA using a tungsten (“Standard”) target. 1440 projection images were taken during a 360° rotation at a total duration of 120 minutes. The 16-bit data set generated by reconstruction (voxel size 0.769 µm) was cropped and converted to 8 bit using VGStudio MAX 2.2 software (Volume Graphics, Heidelberg, Germany). Further visualization procedures were carried out with Amira 6.4 software (Thermo Fischer Scientific, Electron Microscopy Solutions, Hillsboro, Oregon, USA) applying manual segmentation for discrimination of external and internal shell structures. Final visualization was conducted using the Volume Rendering module.

**Scanning Electron Microscopy (SEM-EDX).** SEM-EDX: Sections of the shell of the intact syntype *Z.schaufussi* (NHMW 71837) (SEM) and the elemental composition (EDX) of sediment encrusted on the shell were assessed using the FEI-ASPEX EXpress scanning electron microscope system (Pittsburgh, PA, USA), implementing a BE-detector for image generation. The section of cardboard on which the shell was glued, was mounted on a computer-controlled stage for scanning. Elemental composition was detected (i.e. each element shows a multiple-peak pattern in the spectrum) by using an emission current of 35 μA, an electron beam acceleration voltage of 20 kV under sample pressure of 0.15 Torr and a working distance of 22.4 mm at RJL Micro & Analytic GmbH, Karlsdorf-Neuthard, Germany. Peak height represents the intensity of the element and this is proportional to the mass percentage present in the shell region tested.

## Taxonomy

### Family Carychiidae Jeffreys, 1830

#### Genus *Zospeum* Bourguignat, 1856

##### Zospeum schaufussi

Taxon classificationAnimaliaBasommatophoraCarychiidae

von Frauenfeld, 1862

**Figure 2.**
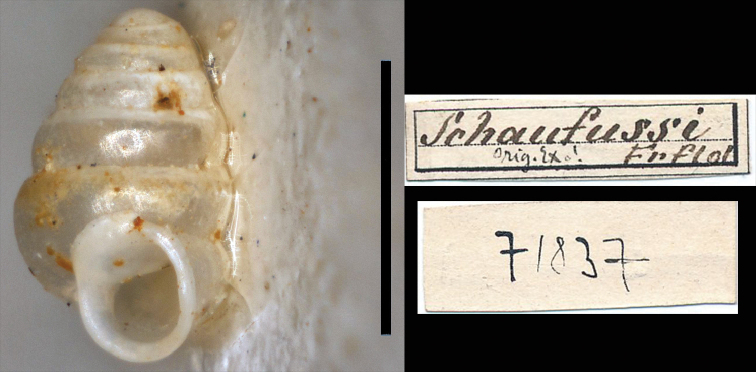
*Zospeumschaufussi* von Frauenfeld, 1862, lectotype and labels (NHMW 71837). Scale bar: 1 mm.

**Figure 3.**
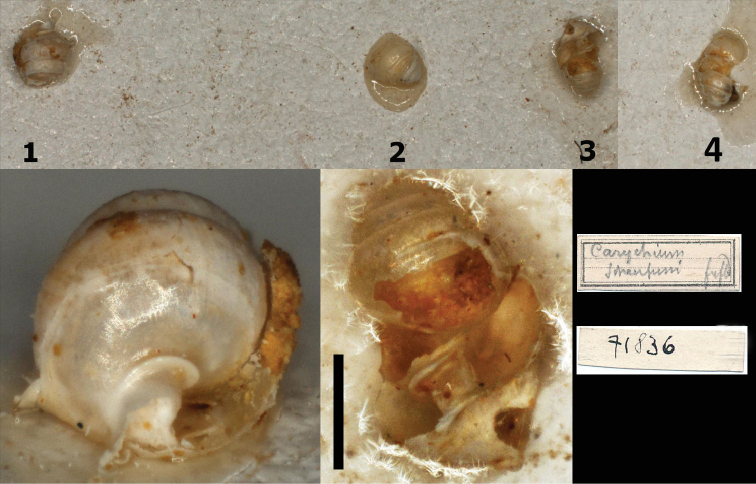
*Zospeumschaufussi* von Frauenfeld, 1862, damaged syntypes and labels (NHMW 71836). Scale bar: 500 μm.

**Figure 4.**
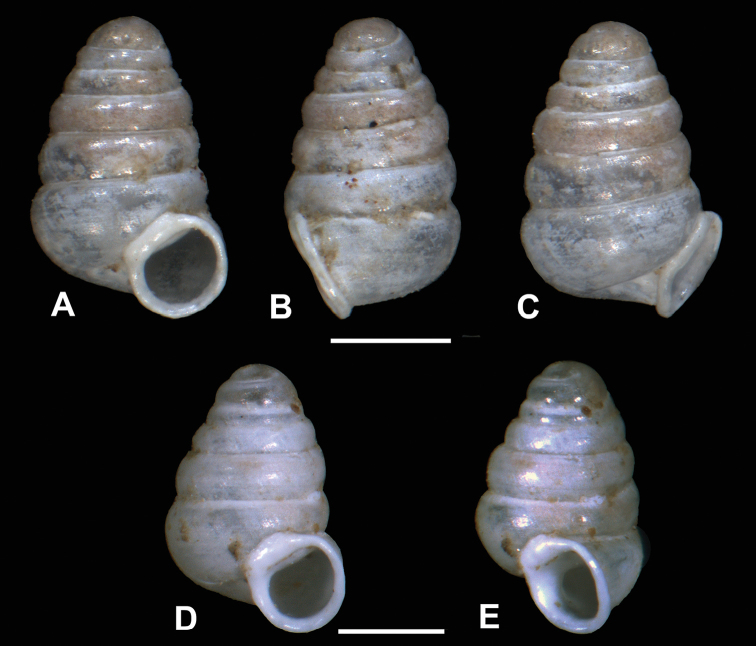
**A–C***Zospeumschaufussi* von Frauenfeld, 1862, Cueva del Búho, Puente Viesgo, Santander, (holotype of *Z.suarezi*, RMNH.MOL.55383) **D–E***Z.praetermissum* sp. n., Cueva del Puente de Inguanzo (RMNH.MOL55389). Scale bar: 500 μm.

Figures 2 , 3 , 4 , 7 , 8 , 9

Zospeum schaufussi von Frauenfeld, 1862.
Zospeum suarezi Gittenberger, 1980: 204. **Syn. n.**

###### Material.

Von Frauenfeld collection, a single undamaged syntype shell (NHMW 71837); 4 broken syntype shells (NHMW 71836). Terra typica: “.. einer neuen Art, welche ich von Hrn. Schaufuss in Dresden erhielt, die darum von Interesse ist, dass er sie in einer Höhle in Spanien auffand, daher die erste Art, welche das geographische Gebiet dieser Gattung mächtig erweitert.” [.. a new species that I received from Mr. Schaufuss in Dresden, which is significant by the fact that he obtained it from a cave in Spain, ... which considerably expands the geographic range of this genus.]. Fischer (1887) narrowed the provenance of *Z.schaufussi* to the greater historical region of Asturias and Cantabria (i.e. Asturia de Oviedo and Asturia de Santillana), but this was apparently overlooked by later authors.

###### Lectotype designation and rationale.

Von Frauenfeld’s (1862) original description [Z. minutissima, vix umbilicata, conica, hyalina, nitida, laeve, anfractibus 5, convexis, apertura rotundata, edentata, peristomate continuo, reflexo] was not detailed enough to recognize the species and lacks an illustration, which in his day, was perhaps deemed unnecessary as no other Iberian congeneric species were known. In Vienna, one of us (AJ) could study five original syntype shells of *Z.schaufussi* (NHMW 71836 – 71837), as was previously done by Gittenberger (1980). The syntypes are firmly glued on two pieces of cardboard (Figs 2–3). Von Frauenfeld mentions that he viewed “some damaged specimens .., without the slightest hint of dentition, such that I cannot doubt the consistent lack of teeth in this species” [translated from German]. All surviving shells, except one (NHMW 71837), are seriously damaged. Gittenberger (1980) concluded that the four damaged syntype shells could not be *Z.schaufussi* because internal barriers are clearly discernible and that the syntypes of the true, edentate, *Z.schaufussi* were missing or lost. We can confirm Gittenberger’s observation of the damaged syntypes (see Fig. 3). Gittenberger (1980) attributed the single undamaged syntype shell to his new species, *Z.suarezi* (as a paratype), rather than to *Z.schaufussi*. We cannot concur with his view. Von Frauenfeld (1862) stressed the similarity with *Z.amoenum* von Frauenfeld, 1856, as the only other toothless *Zospeum* species; in fact, all other *Zospeum* species known by the end of the 19^th^ Century have apertural teeth conspicuously present in frontal view, but the deeper, internal dentition, was often unknown or not specifically addressed in descriptions (see e.g. Kobelt 1899, pls 218–219). We therefore, assume that von Frauenfeld referred to the externally visible dentition in the “apertura”; “Mündung”. The apertural dentition in the intact syntype shell is not, or barely, visible externally (Fig. 2). We conclude that this shell, bearing the label notation “Orig [inal] Ex [emplar]!” (Fig. 2), is the only remaining undamaged syntype of *Z.schaufussi* and thus, designate it here as the lectotype. The purpose of this lectotype designation is the fixation of a taxon name to a specific morphology and to stabilize nomenclature rather than reconstructing the historical course of events.

###### Lectotype description.

Shell minute, ca. 1.3 mm, elongate-conical, with at least 5½ regularly coiled, convex whorls, suture deep; teleoconch smooth; aperture roundish-lunate; peristome thickened, elongate-roundish (not angular), closely adhering to spire; peristome height ca. 36% of shell height; umbilicus closed, umbilical depression deep, wrinkles behind apertural lip leading to umbilicus (seen in SEM-EDX Fig. 9B). Externally, no apertural dentition is visible apart from a rather low lamella (appearing as a barely visible denticle) in the parietal-columellar corner, discernible only in a rather oblique apertural view. Internally, the columella appears as a short, slightly twisted stem, compressed-dilated at its base (Fig. 7C–F), circumscribed by a conspicuous, inclinate lamella that changes in extension along its course. In addition, there may be a hint of secondary lamellar growth at the base of the penultimate whorl (Fig. 7E).

*Zospeumschaufussi* is easily separable from *Z.biscaiense* and *Z.zaldivarae* in shell and peristome shape and apertural characters, whereas *Z.percostulatum* is distinctly ribbed. *Zospeumvasconicum* and *Z.bellesi* are more similar, but the latter has no apertural barriers or even a suggestion of any. The former has a much less prominent columellar lamella and is clearly less tightly coiled. The species described here as *Z.gittenbergeri*, differs by its angular rather than rounded peristome and slightly developed lamella on the columella.

Clearly, the lectotype of *Z.schaufussi* strongly resembles Gittenberger’s topotypic *Z.suarezi*, to the extent that Gittenberger (1980) considered the lectotype shell a paratype of his species. The shell described below as *Z.praetermissum* sp. n., is distinct in its less elongate shell with less tightly coiled whorls and the presence of a second lamella on the base of the columella (Fig. 11G, I, K). Shells of *Z.suarezi* from the type cave agree with the *Z.schaufussi* lectotype in their elongate-conical shell and coiling tightness (see Table 1), rounded peristome, and barely visible dentition in the aperture. Internally, they have a similar columellar lamella configuration.

###### Remarks.

Although our SEM-EDX analyses revealed no significant evidence linking the lectotype to a specific cave or potential cave region, this method, however, revealed some ecological information derivable from the sediment encrusting the shell. The sediment reflects a granitic context and minerogenetic processes (Onac and Forti 2011) acting in the cave environment. Detectable, are different concentrations of calcium (Ca), aluminum (Al), silicon (Si), magnesium (Mg), oxygen (O), carbon (C), iron (Fe), potassium (K), phosphor (P) and lead (Pb).

##### Zospeum praetermissum

Taxon classificationAnimaliaBasommatophoraCarychiidae

Jochum, Prieto & De Winter sp. n.

http://zoobank.org/3B97291A-8B05-41BA-96B1-8F6B50C22B69

**Figure 5.**
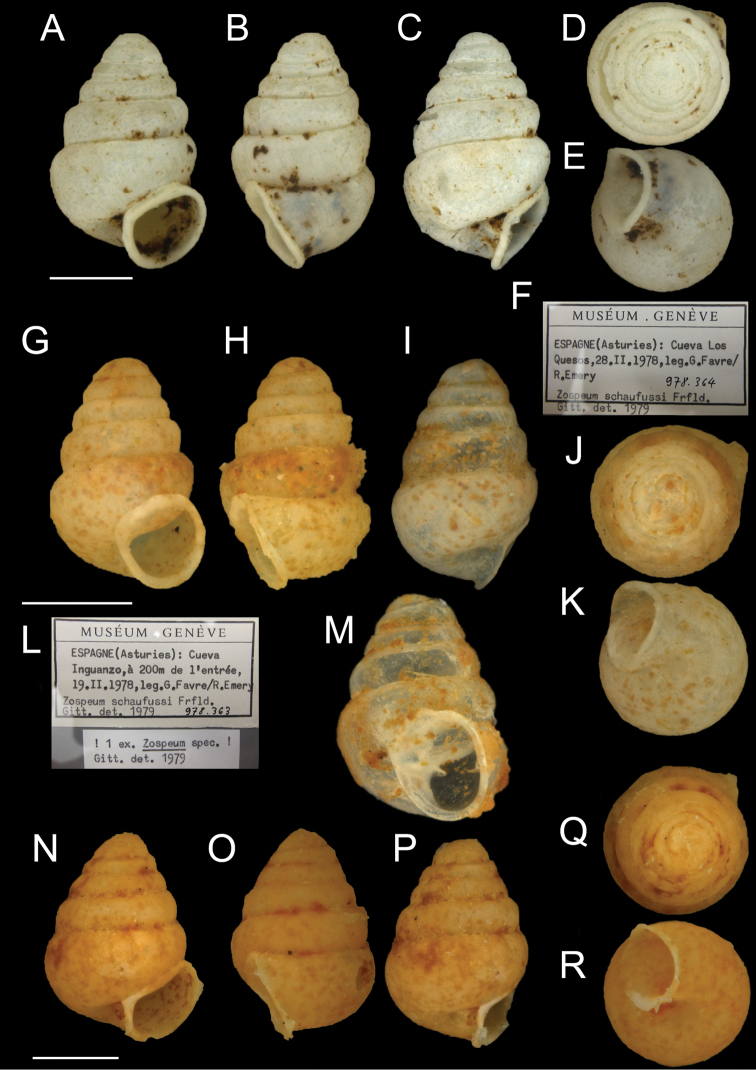
*Zospeum* material assessed in Gittenberger (1980). **A–F***Zospeumpraetermissum* sp. n. (paratype of *Z.suarezi*, MHNG-Mol 96220/1 shell, now lost), Cueva Los Quesos (showing ambivalent label, *Z.schaufussi* Frfld) **G–M***Zospeumpraetermissum* sp. n (figured paratype of *Z.suarezi*, MHNG-Moll 96219, now lost), Cueva del Puente de Inguanzo **L, N–R***Zospeumgittenbergeri* sp. n. (figured shell of *Z.schaufussi* sensu Gittenberger (1980), MHNG 96219, now lost).

Figures 5G–M , 10 , 11

Zospeum suarezi — Gittenberger 1980: 203, Fig. 2 (only shells from Cueva de Inguanzo).
Zospeum suarezi — Gómez and Prieto 1983: 8, Fig. 1 (only the named shell).

###### Type Material.

**Holotype.** SPAIN Cueva del Puente de Inguanzo, Inguanzo, Concejo de Cabrales, Asturias, MGRS 30TUN4897097640; N43.315574, W4.860905; 230 m a.s.l.; 19 Feb 1979; G. Favre & R. Emery leg.; RMNH.MOL.55391 [55386]. **Paratypes.** SPAIN locus typicus: 2 shells; data as the holotype; RMNH.MOL.339954 [55386]. **Other material.**MHNG 96219/1 shell (now lost).

###### Diagnosis.

Shell ca. 1.1–1.2 mm, conical with a roundish and moderately thick peristome, lacking apparent apertural barriers but with a small distinct lamella (denticle) in the parietal-columellar corner; internally, columella robust with a central lamella, a low upper columellar bulge and a basal umbilical ridge.

###### Description.

Measurements of holotype and paratypes are presented in Table 1. Shell minute, fresh shells transparent, variable in shape (SH:SD ratio 1.41–1.57) with ca. 5 whorls, regularly coiled, suture deep, whorls convex, more or less strongly shouldered; teleoconch sculpture consists of occasional blunt growth lines; robust columella with an inclinate, central lamella (Fig. 11F); an upper lamellar bulge swells from the base of the inner penultimate whorl above the central lamella (Fig. 11G, I, K); a distinct, short, ridge projects from the base of the columella above the umbilical indentation (Fig. 11G, I, K); aperture more or less circular; peristome adhering to spire, reflected, moderately thickened, roundish; umbilicus closed; apertural barriers absent apart from the low lamella that appears externally as a small, but distinct denticle on the parietal-columellar corner visible in oblique apertural view (Figs 5M, 10D).

###### Differential diagnosis.

Differs from *Z.schaufussi* externally by its more conical shell with less tightly coiled whorls – adult shells having around 5 rather than 6 whorls of the same size – and a more pronounced lamella/denticle visible in the parietal-columellar corner in oblique view; internally by its robust columella and by its pronounced basal columellar ridge (above umbilical indentation). *Zospeumpraetermissum* is easily distinguished from *Z.biscaiense* and *Z.zaldivarae* in shell and peristome shape and apertural characters, whereas *Z.percostulatum* is distinctly ribbed. *Zospeumgittenbergeri* differs by its broad, angular peristome and barely developed lamella on the columella. *Zospeumvasconicum* has a more rounded aperture with an almost uniformly-thickened peristome. *Z.bellesi* has no apertural barriers nor a columellar lamella.

###### Etymology.

The name, *praetermissum*, refers to the situation that the holotype shell was originally not recognized as distinct from *Z.suarezi*.

###### Distribution.

Only known from the type locality.

###### Ecology.

According to the records of the speleologist Gérald Favre (pers. comm. 2017), the collection site was located ca. 200 m from the cave entrance (also noted on the data label NHMG-Moll 96219 (978.363)). His field notes document that the collection area consisted of sandy substrate, some chestnut fragments and that the humidity level was low. The total length of the cave is 1500 m.

###### Remarks.

**Figure 6.**
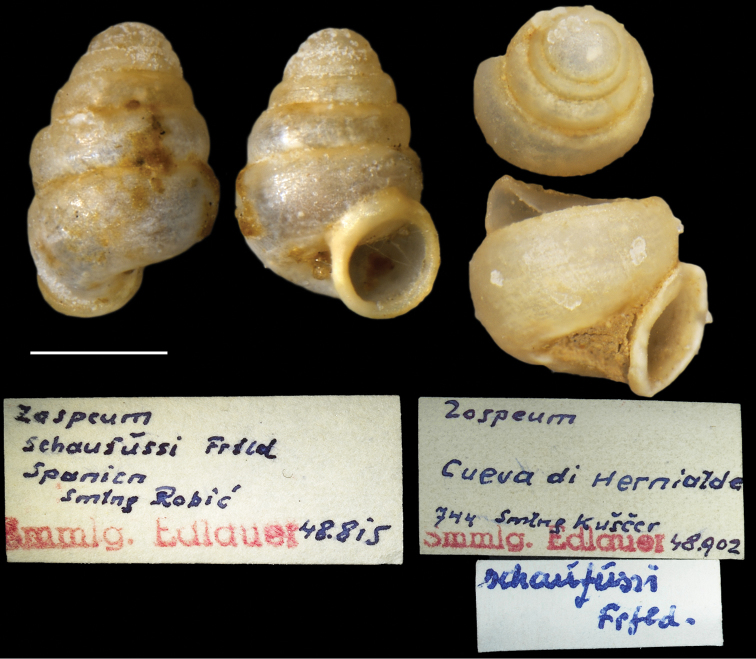
Zospeumcf.vasconicum Prieto, De Winter, Weigand, Gómez & Jochum, 2015. Ex. *Z.suarezi* paratype, NHMW-MO 75000-E-48815, Cueva Hernialde, Guipuzcoa, assessed in Gittenberger (1980) („NWW-Coll. Edlauer 48815, ex. Coll. Robić/1shell”).

The label to the shell material Gittenberger (1980) assigned as *Z.suarezi* paratypes (NHMG-Moll.96219/1 shell), bore Gittenberger’s apparent ambivalence “Zospeum schaufussi Frfld Gitt. det. 1979” (Fig. 5L). Although currently lost, this shell was most likely conspecific with *Z.praetermissum*. We consider the paratype *Z.suarezi* shell (NHMW-MO 75000-E-48815) from Cueva Hernialde, Guipuzcoa to be Z. cf.vasconicum (Fig. 6).

##### Zospeum gittenbergeri

Taxon classificationAnimaliaBasommatophoraCarychiidae

Jochum, Prieto & De Winter sp. n.

http://zoobank.org/6506C1D6-746D-4ABE-B43E-6D51FCEAEF33

Figures 5L, N–R , 13 , 14

Zospeum schaufussi —Gittenberger 1980: 203, Fig. 1. [**non**Zospeumschaufussi von Frauenfeld, 1862]

###### Type material.

**Holotype** (RMNH 234166/1 shell): Cueva del Puente de Inguanzo (Inguanzo, Concejo de Cabrales, Asturias, Spain), MGRS 30TUN4897097640 (N43.315574, W4.860905), 230 m a.s.l., 19.02.1979, leg. G Favre & R Emery. **Other material**: former *Z.suarezi* paratype shells mentioned in Gittenberger (1980): data as holotype: MHNG 96219/4 shells (now lost) (Fig. 5L, N–R).

###### Diagnosis.

Holotype shell conical, larger than most Iberian *Zospeum*, SH nearly 1.5 mm with 5 1/2 moderately convex whorls. Parietal part of peristome straight and long, giving the peristome an angular rather than convex appearance. Internally, the lamella circumscribing the columella is very weak, but it is unclear if this is due to erosion or if it is covered by debris (Fig. 14G–J).

###### Description.

Measurements of holotype provided in Table 1. Lost specimen of imaged MNHG 96219 shell (Fig. 5N–R) is smaller than holotype (SH 1.22 mm). Shell elongate-conical with approximately 5 ½ rounded whorls, regularly coiled, suture deep; teleoconch smooth with occasional blunt growth lines (Fig. 14A–D); aperture more or less circular; peristome closely adhering to spire, reflected, moderately thickened, with an angular, relatively long parietal callus; columella straight and aligned axially, single lamella small and non-extending, coiled tightly around the columella; columellar apertural edge (side view, aperture facing right) and the border of the parietal callus join at an angle of ca. 90 degrees (Fig. 13C); umbilicus closed, umbilical depression deep, moderately strong, wrinkly striae descend from last whorl behind apertural lip leading to umbilicus; apertural barriers lacking.

**Figure 7.**
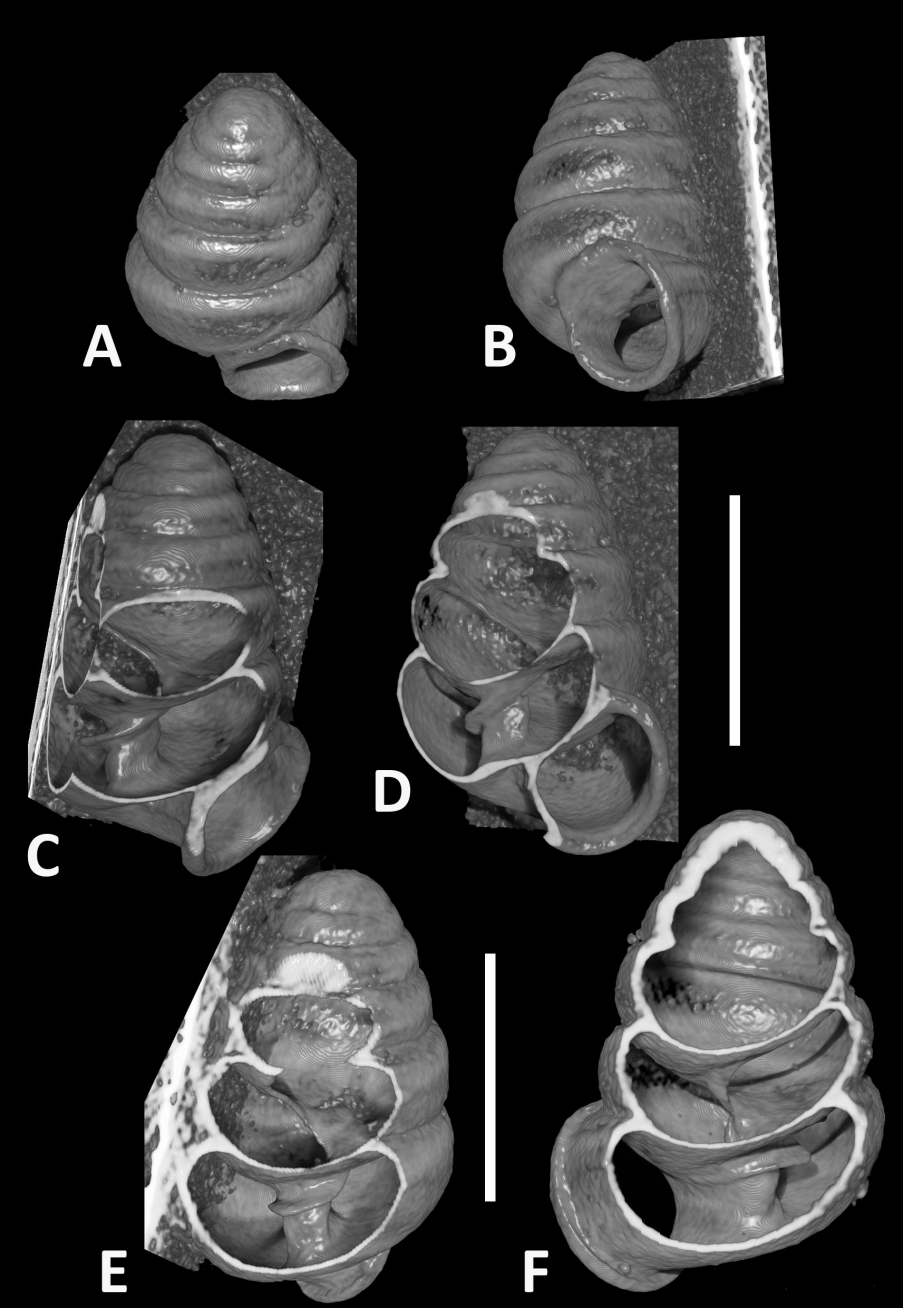
CT images showing columellar apparatus of *Zospeumschaufussi* von Frauenfeld, 1862, lectotype (NHMW 71837). Scale bar: 500 μm.

**Figure 8.** Animated video from CT scans of *Zospeumschaufussi* von Frauenfeld, 1862, lectotype (NHMW 71837). Scale bar: 500 μm.

###### Differential diagnosis.

Differs from *Z.biscaiense* by the larger, more elongate shell and the absence of major apertural barriers; from *Z.schaufussi* (lectotype) by its long and angular parietal callus, straight, axially aligned columella and its small, non-extensive, tightly coiled lamella; in *Z.bellesi*, columellar elaboration is completely absent; from *Z.vasconicum* by its non-rounded aperture, more robust, axially aligned columella; from *Z.zaldivarae* by its more elongate shell, lack of apertural barriers; from *Z.percostulatum* by its non-costulate shell; from *Z.praetermissum* sp. n. by its long, parietal edge of the angular peristome and different columellar morphology.

###### Etymology.

The new species is named in honour of Prof. Edmund Gittenberger, in recognition of his pioneering work on Iberian *Zospeum*.

###### Distribution.

Only known from the type locality.

###### Ecology.

According to the records of the collector, Gérald Favre (pers. comm. 2017), the collection site was located ca. 200 m from the cave entrance. His field notes document that the area consisted of sandy substrate, some chestnut fragments and that the humidity level was low.

**Figure 9.**
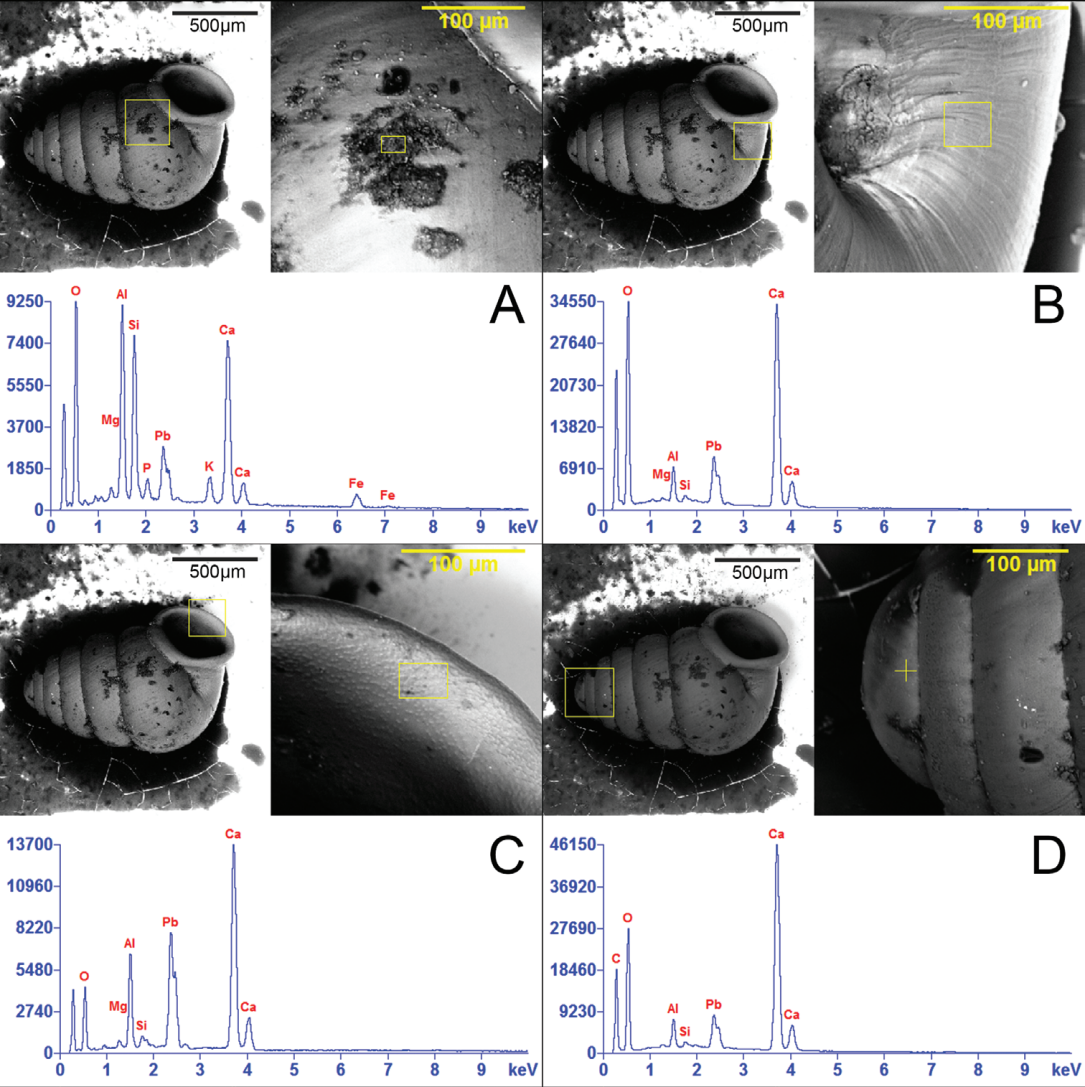
SEM-EDX spectroscopic images showing spectrum of elemental content in sediment encrusted on different regions of the lectotype of *Zospeumschaufussi* von Frauenfeld, 1862 (NHMW 71837). **A–D** Concentrations within yellow-framed zone of calcium (Ca), aluminum (Al), silicon (Si), magnesium (Mg), oxygen (O), carbon (C), iron (Fe), potassium (K), phosphor (P) and lead (Pb).

**Figure 10.**
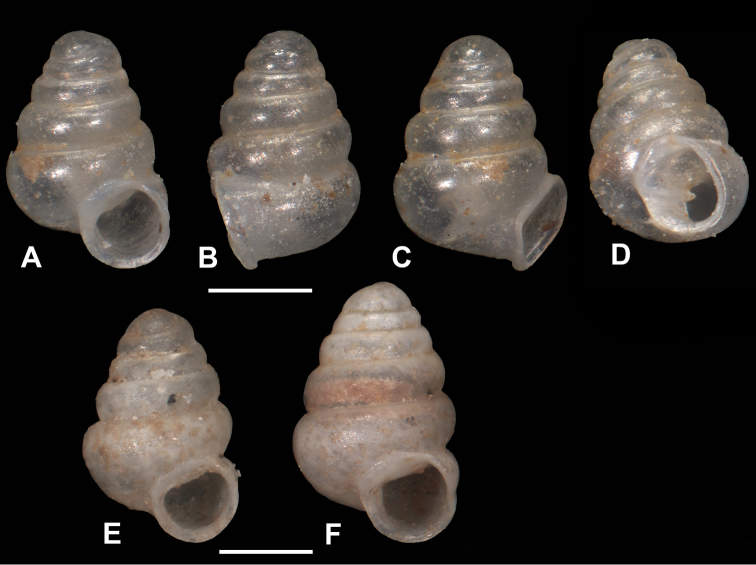
*Zospeumpraetermissum* sp. n. **A–D** holotype (RMNH.MOL.55391), shell illustrated by Gittenberger (1980: fig. 2) **E–F** paratypes (RMNH.MOL.339954). Scale bar: 500 μm.

###### Remarks.

We formally describe the specimen illustrated by Gittenberger as a separate species. We confine the type material to the one shell known from the type cave. Although Gittenberger (1980) mentioned similar, but much less elongate shells from another cave (Cueva del Búho), we prefer to address these as Z.cf.gittenbergeri (Fig. 13 F–I). Shells with this shape and apertural morphology occur in various caves in sympatry with a *schaufussi*-like (in its present sense) species. This is seen for example in shells from Cueva del Linar (C. la Busta), where shells with angular peristomes, but much smaller than the holotype shell (SH 0.95 – 1.15; SD 0.64 – 0.74 mm) occur sympatrically with shells that are externally and internally indistinguishable from *Z.schaufussi*. Internal morphology of the Cueva de Linar shells so far shows a simple, tightly coiled singular lamella around the columella (unpubl. results, Jochum). Further study is needed to define *Z.gittenbergeri*, especially using molecular data.

**Figure 11.**
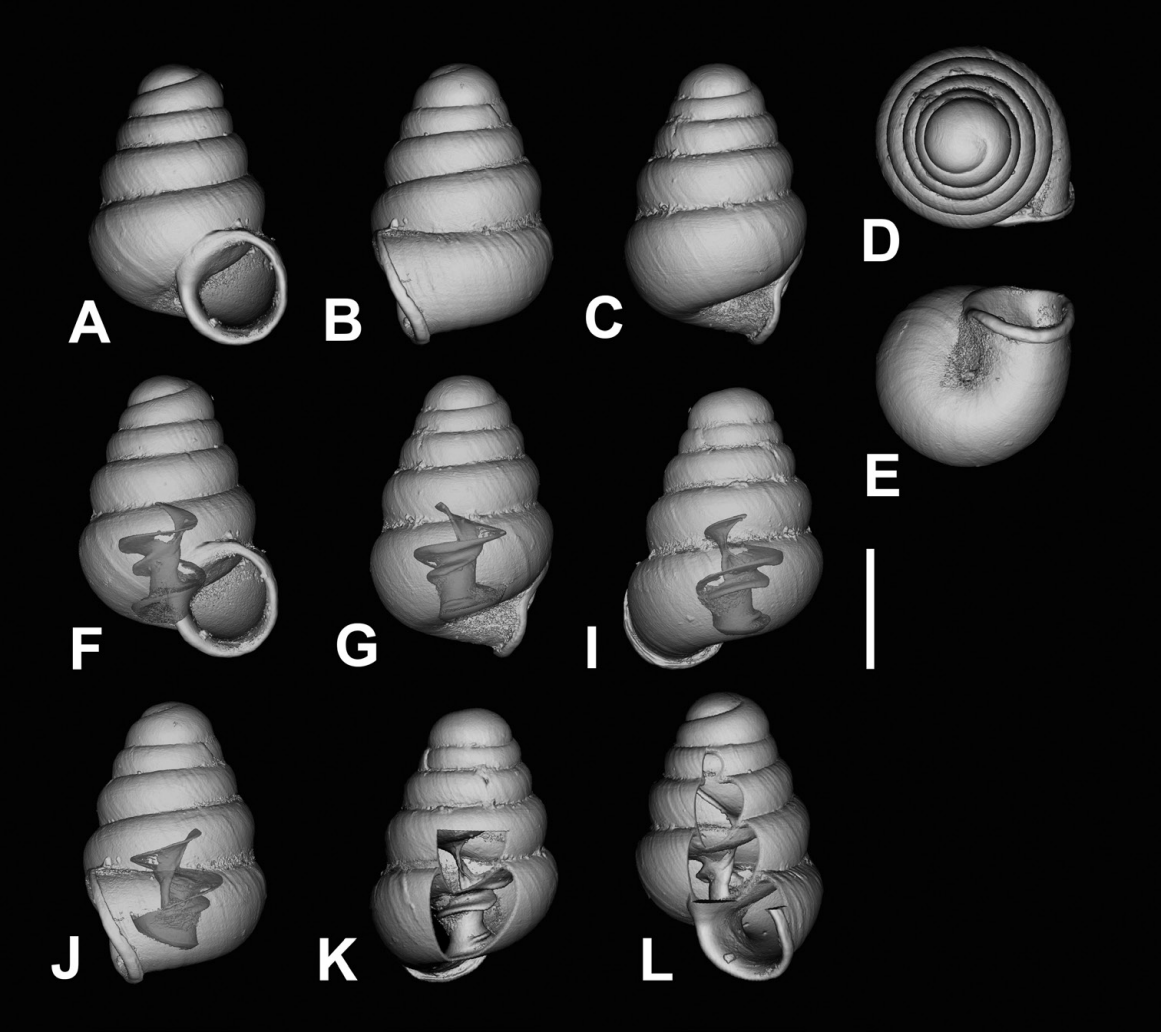
CT images of *Zospeumpraetermissum* sp. n. holotype (RMNH.MOL.55391). **F** inclinate lamella **G, I, K** show upper lamellar bulge, central lamella, and columellar basal ridge. Scale bar: 500 µm.

**Figure 12.**
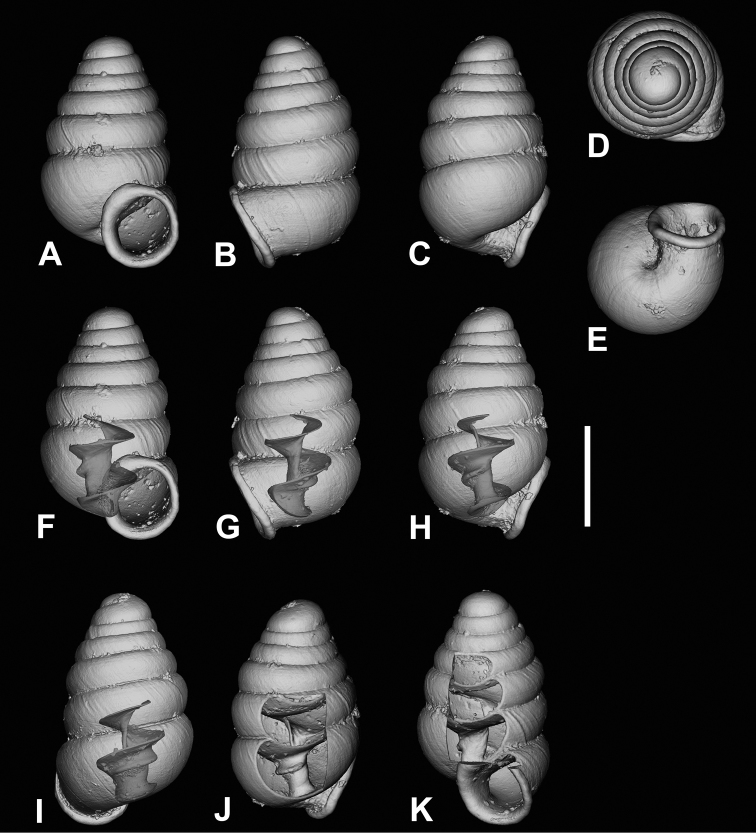
CT images of *Zospeumsuarezi* syn. n. of *Z.schaufussi* von Frauenfeld, 1862, paratype *Z.suarezi* (Gittenberger 1980), RMNH.MOL.55389 from type locality. Scale bar: 500 µm.

A number of confusing discrepancies surfaced in addressing Gittenberger’s (1980) material from Cueva del Puente de Inguanzo. Gittenberger (1980) cited only 1 shell (corresponding to the data of specimen MHNG 96219) for *Z.schaufussi* (= *Z.gittenbergeri* sp. n.), which he measured (1.45 mm) and illustrated (fig.1). For this shell, he cites “Cueva de Inguanzo near Inguanzo, 2 km SW of Cabrales, between Covadonga and Panes, Oviedo; UTM U N 4 9; G. Favre & R. Emery leg., 19.ii.1979 (MHNG/i shell; RMNH/i shell).” However, our Figure 5 (L, N–R) is the shell imaged by the curator, Emmanuel Tardy, at the MHNG (2017) but it only measures 1.22 mm and does not correspond to the illustrated shell. On the other hand, the shell chosen as holotype (RMNH.234166), measures 1.49 mm and fits the one Gittenberger (1980) illustrated. It appears that Gittenberger (1980, fig. 1) erroneously indicated MHNG as the collection provenance of the shell, which is actually, the single shell at the RMNH.

An additional source of confusion is that there are two caves with similar, but not identical names referring to the town of Inguanzo, which are situated within one kilometer of each other (Fig. 15). The type cave, Cueva del Puente de Inguanzo, from where Gittenberger’s (1980) material derives, is a different cave from where Weigand et al.’s (2013) material, from Cueva de Inguanzo (= Cueva de Bosque), derived. These two caves are separated by the Casaño River and it is not known if the two cave systems are contiguous. This important consideration became apparent when we contacted the collector of Gittenberger’s (1980) material.

**Figure 13.**
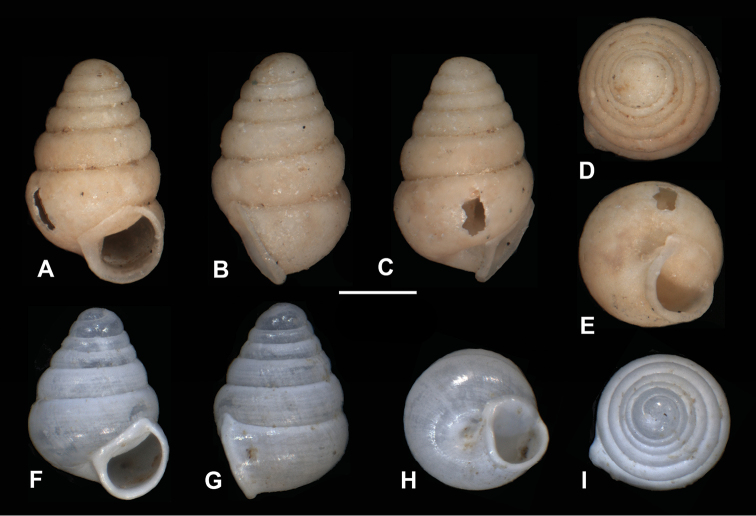
*Zospeumgittenbergeri* sp. n. **A–E** holotype (RMNH.MOL.234166) **F–I**Z.cf.gittenbergeri from Cueva del Búho (RMNH.MOL.234165). Scale bar: 500 µm.

**Figure 14.**
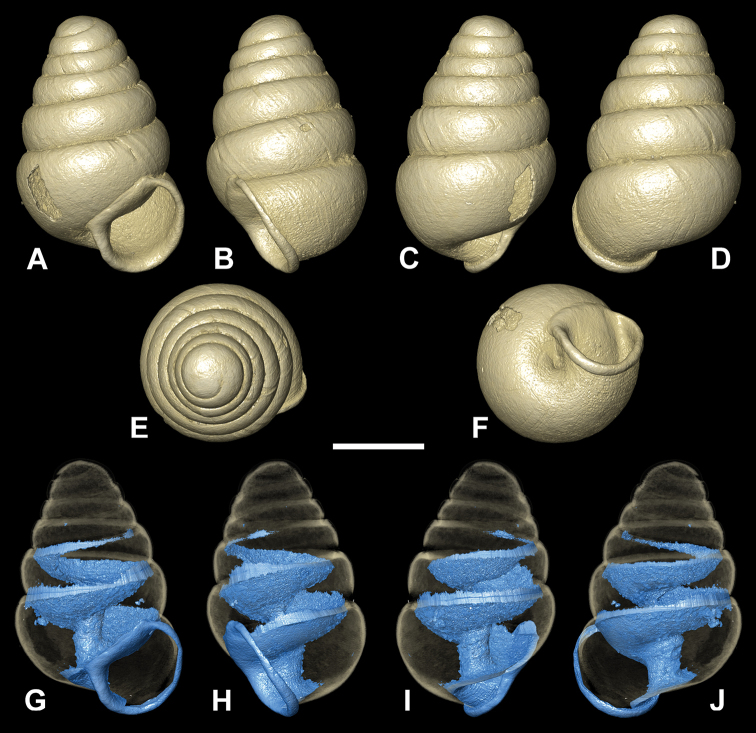
CT scans of *Zospeumgittenbergeri* sp. n. holotype (RMNH.MOL.234166). Scale bar: 500 µm.

**Figure 15.**
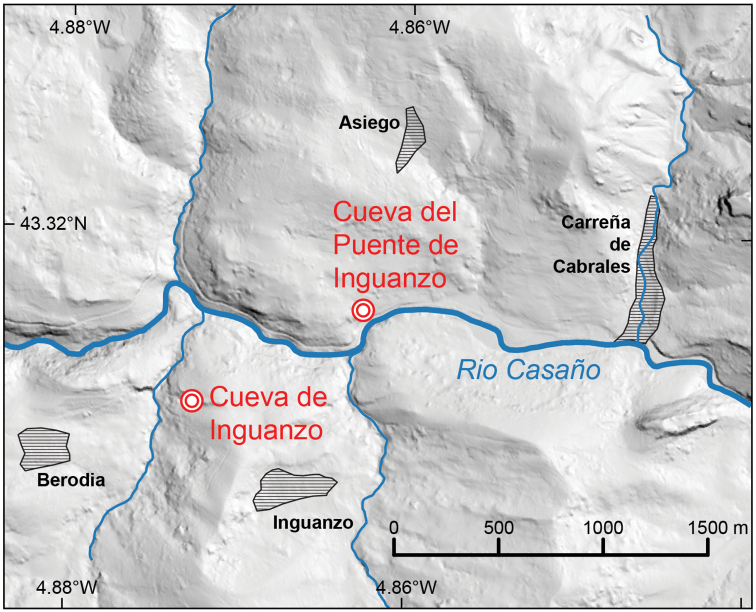
Map indicating geographic position of the two different Inguanzo-named caves on either side of the Rio Casaño: Cueva del Puente de Inguanzo (collection site of Gittenberger’s (1980) shells) and Cueva de Inguanzo (site of Weigand et al. (2013) molecularly-assessed material). Source of DEM data: LiDAR-PNOA DGM 5 m owned by Instituto Geográfico Nacional (IGN) and provided under CC-BY 4.0 license.

## Supplementary Material

XML Treatment for Zospeum schaufussi

XML Treatment for Zospeum praetermissum

XML Treatment for Zospeum gittenbergeri
